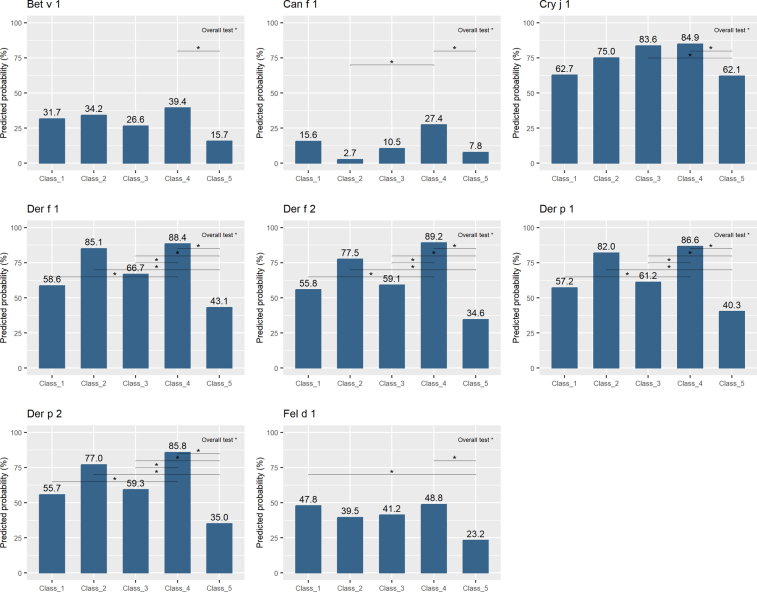# Lung functions and IgE component sensitizations: Five wheezing phenotypes in adolescents from the T-Child study in Tokyo

**DOI:** 10.1016/j.jacig.2025.100480

**Published:** 2025-04-19

**Authors:** Kiwako Yamamoto-Hanada, Limin Yang, Mayako Saito-Abe, Fumi Ishikawa, Miori Sato, Yumiko Miyaji, Motoko Mitsui-Iwama, Yusuke Inazuka, Koji Nishimura, Kenji Toyokuni, Hiroya Ogita, Tomoyuki Kiguchi, Yoshitsune Miyagi, Shinichiro Inagaki, Shigenori Kabashima, Tatsuki Fukuie, Masami Narita, Elizabeth Huiwen Tham, Yukihiro Ohya

**Affiliations:** a Allergy Center, National Center for Child Health and Development, Tokyo, Japan; b Medical Support Center for the Japan Environment and Children’s Study, National Research Institute for Child Health and Development, Tokyo, Japan; c Department of Paediatrics, Yong Loo Lin School of Medicine, National University of Singapore (NUS), Singapore; d Khoo Teck Puat-National University Children’s Medical Institute, National University Health System (NUHS), Singapore; e Department of Occupational and Environmental Health, Graduate School of Medical Sciences, Nagoya City University, Aichi, Japan; f Division of General Allergy, Bantane Hospital, Fujita Health University, Aichi, Japan

**Keywords:** Wheezing, phenotype, lung function, Feno, sensitization, birth cohort

## Abstract

**Background:**

The endophenotypes of allergic disorders are known to vary across racial groups, underscoring the importance of studying allergic disease phenotypes in the population of Tokyo.

**Objective:**

We sought to elucidate the developmental trajectories of wheezing among adolescents and their associations with pulmonary function and IgE sensitization in the Japanese pediatric population.

**Methods:**

The research used data from the Tokyo Children’s Health, Illness, and Development study, a comprehensive Tokyo birth cohort study, which recruited 1701 mother-infant dyads prenatally and followed the children up from birth till age 13 years. The analytical approach was conducted in 4 distinct phases: (1) delineation of trajectory groups using latent class growth analysis; (2) detailed characterization of each identified trajectory; (3) assessment of the relationships between predictors and wheezing groups through multinomial logistic regression; and (4) examination of the interrelations among trajectory groups, lung function, and IgE sensitization at age 13 years.

**Results:**

A total of 5 unique wheezing phenotypes were discerned: early-onset transient wheezing (10.2%), late-onset transient wheezing (7.3%), low frequent wheezing (15.0%), persistent wheezing (11.9%), and never/infrequent wheezing (55.5%). No statistically significant deterioration in impulse oscillometry parameters and spirometry parameters except %V25 was detected across any of the phenotypes. Nonetheless, the persistent wheezing phenotype demonstrated an association with lowered %V25, elevated fractional exhaled nitric oxide levels, and an increased prevalence of sensitization to multiple allergens at age 13 years.

**Conclusions:**

The wheezing phenotypes identified in this study displayed distinct characteristics. Importantly, despite the diverse wheezing trajectories observed from birth, adolescents in Tokyo did not exhibit any discernible decline in lung function.

Asthma constitutes a prevalent chronic inflammatory respiratory condition affecting both pediatric and adult populations, with wheezing recognized as one of its hallmark manifestations. Asthma in adolescents presents a significant global public health concern, characterized by considerable fluctuations in prevalence and associated risk factors contingent upon geographic regions.^1^ The Global Asthma Network Phase I investigation, encompassing a cohort of 157,784 adolescents across 25 nations, documented the prevalence of asthma symptoms to be 11% among individuals aged 13 to 14 years.^2^ A governmental surveillance initiative conducted in Japan disclosed that in the year 2017, there were 1794 fatalities attributable to asthma, with no recorded deaths in the pediatric group aged 0 to 14 years and a solitary death among those aged 15 to 19 years.^3^ The mortality rate associated with asthma for the pediatric population aged 0 to 4 years has consistently declined since 1950, remaining notably low at 0 to 0.1 per 100,000 individuals since 2008.^3^

Past birth cohorts from Europe, the United States, and Australia have delineated the correlations between wheezing phenotypes and compromised lung function, heightened fractional exhaled nitric oxide (Feno) levels, and IgE sensitization among adolescents.^4^, ^5^, ^6^, ^7^, ^8^ In previous investigations, we described 5 distinct wheezing phenotypes observed in a Tokyo birth cohort up to age 9 years.^9^ A Singapore birth cohort identified 4 different wheezing phenotypes extending to age 8 years.^10^ Nevertheless, there is a paucity of literature, particularly in the Asian adolescent population, regarding wheezing phenotypes in association with lung function, airway inflammation, and IgE sensitization. It is acknowledged that the phenotypes and endotypes associated with allergic disorders exhibit variability across racial groups, underscoring the importance of dedicated research within Asian populations. Comprehensive studies across diverse populations and environmental contexts are essential to deconvolute the heterogeneous characteristics of these conditions. We hypothesized that wheezing phenotypes from birth to adolescence might be associated with impaired lung function and IgE sensitization in children in Tokyo. This investigation sought to analyze the trajectory of wheezing throughout adolescence and its association with lung function and IgE sensitization among adolescents in Tokyo.

## Methods

### Study design

**Supplementary Fig E1:**
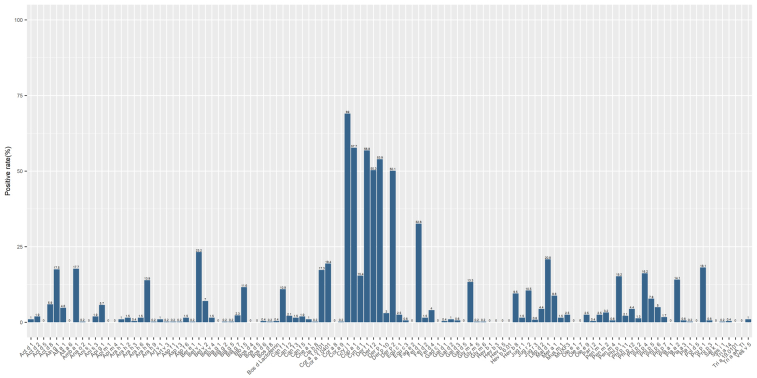


This study used data from the birth cohort of the Tokyo Children’s Health, Illness, and Development (T-Child) study,^11^ one of the Asia Allergy Birth Cohort (A2BC) network initiatives.^12^ This cohort recruited 1701 pregnant women, who attended their first prenatal visit at the National Center for Child Health and Development before 16 weeks of gestation between October 2003 and December 2005, and followed up their 1550 newborns from birth. The study collected data from birth to adolescence through repeated questionnaire surveys, medical examinations, blood tests, lung function tests, and other approaches. Detailed descriptions of the study methodology have been published previously.^9^^,^^13^, ^14^, ^15^, ^16^ In this article, we provide data extracted from questionnaire surveys administered during pregnancy and postnatally up to age 13 years as well as allergen-specific IgE and lung function test data of the children at age 13 years. We excluded those missing IgE or lung function test data at age 13 years. After data selection, we obtained 475 cases for the present analysis. The study flow chart is shown in Fig E1 (in the Online Repository available at www.jaci-global.org).

This study adhered to the principles of the Declaration of Helsinki and complied with national regulations and guidelines. The T-Child study received approval from the National Center for Child Health and Development Research Ethics Committee (approval no. 52) and followed the Japanese ethical guidelines for medical research on humans along with the Helsinki Declaration. Informed written consent was obtained from all parents at recruitment and before conducting the questionnaire surveys and study measures for their children. In addition, informed written assent was obtained from all adolescent participants before they took part in the questionnaire surveys and study measures.

### Wheezing, atopic dermatitis, and rhinitis episodes

We examined wheezing, atopic dermatitis, and rhinitis episodes from the International Study of Asthma and Allergies in Childhood (ISAAC) questionnaires administered in children from age 1 to 13 years. The questionnaire defined current wheeze as a positive response to the question “Has your child ever had wheezing or whistling in the past 12 months?” We obtained wheezing data at 12 time points (children aged 1-9 and 11-13 years).

### Lung function and serum allergen-specific IgE at 13 years

We performed lung function tests for all children at age 13 years (spirogram, impulse oscillometry [IOS] system by MasterScreen IOS-J, FUKUDA SANGYO Co, Ltd, Tokyo, Japan^17^ and Feno testing by NIOX VERO, Chest Co, Ltd, Tokyo, Japan^18^). We performed spirometry, IOS, and Feno testing according to the *Handbook of Pediatric Pulmonary Function Tests*^19^ and assessed various parameters. We evaluated serum allergen-specific IgE levels at age 13 years by ISAC ImmunoCAP (cutoff value, 0.3 [≥0.3; <0.3]).^11^

### Covariates for trajectories and other variables

The covariates for trajectories and other variables were collected from questionnaires. These included sex, parental history of wheeze, smoking exposure at 6 months, income during pregnancy, pet ownership at 6 months, maternal age, and maternal education level, which was categorized into 2 groups: low education level (junior high school/high school) and others (vocational school, junior college, university, and graduate school).

### Statistical analysis

We used data from 12 time points between age 1 year and 13 years (1-9 and 11-13 years) and the latent class growth analysis (LCGA) model to classify wheezing trajectories.^20^^,^^21^ In LCGA, variation between individuals is not allowed within groups. Because wheeze is a binary variable, we specify binary logit distributions to estimate the maximum likelihood function. Data were assumed to be missing at random; missing values were handled by the full information maximum likelihood using all available information in all observations. The start value was set as 500.

We decided on the number of groups in LCGA on the basis of the statistical indices of the model, the size of each group, and the interpretability of the group. The statistical indices of the model included the Akaike information criterion, the Bayesian information criterion, the sample size–adjusted Bayesian information criterion, entropy, the Vuong-Lo-Mendell-Rubin likelihood ratio test, the Lo-Mendell-Rubin likelihood ratio test, and the bootstrapped likelihood ratio test. For statistical indicators, small values of the first 3 criteria represented the model to be better. The entropy value was between 0 and 1, and values of 0.4, 0.6, and 0.8 indicated poor, medium, and good classification, respectively. We also computed the average posterior probability by an assigned group on the basis of the maximum posterior probability rule. A high degree of agreement indicated a good classification.

The model selection process was based on the recommendations by Nagin^21^: first, deciding on the number of groups, followed by searching the order of the polynomial and determining the shape of trajectories given the decided number of groups.^21^ Because specifying a cubic trajectory would cause the model to fail to converge, we specified that all trajectories were quadratic, and so we looked for the most appropriate model for 1 to 6 groups. When the number of groups was decided, we specified a class as linear on the basis of the experience of similar related studies and again searched for an optimal model.

The trajectories were plotted according to the following calculation of probability of wheezing^21^:

(1) $$P\left(\text{wheezing}=1\right)=\frac{e^{y^{*}}}{1+e^{y^{*}}}\text{,}$$

(2) $$y^{*}=i+s*\left(\text{scaled}\;\text{age}\right)+q*{\left(\text{scaled}\;\text{age}\right)}^{2}-threshold\text{.}$$

The posterior probabilities of group membership were then calculated^21^:

(3) $$\hat{P}\left(j|Y_{i}\right)=\frac{\hat{P}\left(Y_{i}|j\right){\hat{\pi }}_{j}}{\sum_{j}^{J}\hat{P}\left(Y_{i}|j\right){\hat{\pi }}_{j}}\text{,}$$

where $\hat{P}\left(Y_{i}|j\right)$ is the estimated probability of $Y_{i}$, assuming membership in group *j*, and ${\hat{\pi }}_{j}$ is the estimated probability of membership in group *j*. The maximum estimated posterior probability determined the class assignment.^21^

To explain the classification, we listed the distribution of individual characteristics, including sex, smoking exposure, parental history of wheezing, pet ownership, atopic dermatitis, rhinitis, and IgE sensitization at 5, 9, and 13 years, within each group to provide profile information of the latent classes. We further explored the relationship between covariates and trajectory membership by fitting a multinomial logistic regression model for k classes. Covariates in the model included sex, parental history of wheezing, smoking exposure, low income, and pet ownership. The coefficient of covariate was the increase in the log odds of being in the k class versus the reference class for a unit increase in covariate. Because data with any missing value on covariates would be deleted, we used multiple imputation to deal with missing data on covariates. Variables used for multiple imputation include sex, parental history of wheezing, smoking exposure, low income, pet ownership, maternal age, and maternal education level. We first generated 50 imputed data sets and then used them to obtain pooled parameters by fitting the LCGA model with an auxiliary approach (the R3STEP^22^ in Mplus).

Finally, we evaluated the relationship between trajectory groups obtained earlier and lung function and serum allergen-specific IgE levels at age 13 years. For continuous distal outcomes, the Bolck-Croon-Hagenaars (BCH) procedure^22^ in Mplus was used to estimate the mean parameters of distal outcome for each latent class. For categorical distal outcomes, probabilities of distal outcomes across classes were calculated by the DCAT procedure^22^ in Mplus. A Wald test for the overall test of association and a paired-wise test were performed.^23^ The *P* values from significant tests were adjusted using the Benjamini-Hochberg method.

Mplus 8.11 and R 4.3.2 (R Development Core Team, Vienna, Austria) were used for analysis.

## Results

### Descriptive statistics

The baseline characteristics of the participants are provided in Table E1 (in the Online Repository available at www.jaci-global.org). At age 6 months, the prevalence of passive smoking exposures was 30.28% and that of pet ownership was 8.74%. Only 6.38% of participants were from low-income families. Participants included in this study tended to have higher rates of advanced pregnancy (*P* = .03) and smoking exposure (*P* = .01) compared with participants who were excluded (see Table E2 in this article’s Online Repository at www.jaci-global.org). In the 13-year-olds, sensitization rates of greater than 50% included Der p 2 (50.1%), Der f 2 (50.3%), Der p 1 (53.9%), Der f 1 (56.8%), Cup a 1 (57.7%), and Cry j 1 (69%) (Fig E1). Table E3 (in the Online Repository available at www.jaci-global.org) presents the median and interquartile range for spirometry and IOS variables at age 13 years.

### Fitting LCGA for wheezing type and assessing the model fit

**Fig 1:**
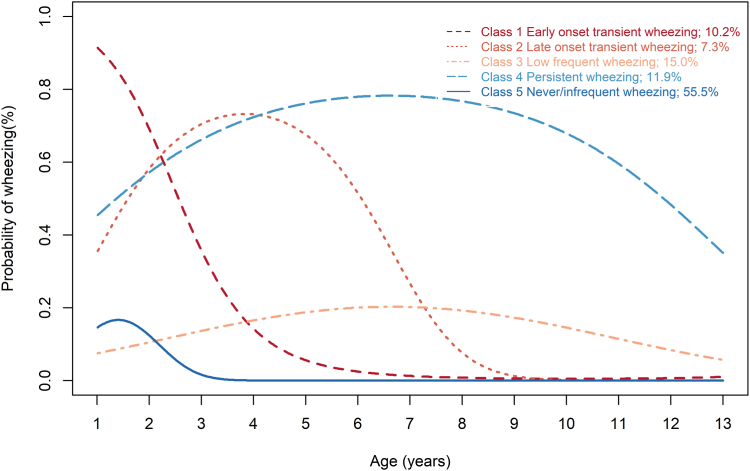
Trajectories of wheezing in childhood. The LCGA model was used to classify trajectories of wheezing from age 1 to 13 years. Five classes were identified as follows: early-onset transient wheezing, late-onset transient wheezing, low frequent wheezing, persistent wheezing, and never/infrequent wheezing. The group membership probabilities for the classes were 10.2%, 7.3%,15.0%,11.9%, and 55.5%, respectively.

**Supplementary Fig E2:**
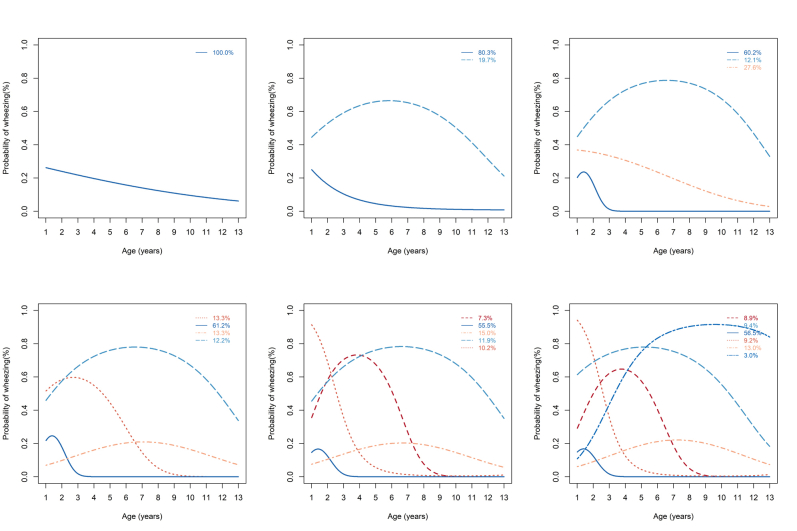


Table E4 and Fig E2 (in the Online Repository available at www.jaci-global.org) show the statistical indices for 1- to 6-class models for wheezing at ages between 1 and 13 years. Although the smallest Bayesian information criterion was found in the 6-class model, because of the presence of very small groups (membership probability, <5%) in this model, we finally chose the 5-class model with all classes specified as quadratic as the most appropriate model (Fig 1). According to the shapes of trajectories, we named the 5 classes as follows: class 1, early-onset transient wheezing (10.2%); class 2, late-onset transient wheezing (7.3%); class 3, low frequent wheezing (15.0%); class 4, persistent wheezing (11.9%); and class 5, never/infrequent wheezing (55.5%) (Fig 1). Table E4 lists the average posterior probabilities by assigned groups on the basis of the maximum posterior probability rule. The average latent class probabilities (see Table E5 in this article’s Online Repository at www.jaci-global.org) ranged from 0.77 to 0.91, implying an accurate classification.

### Profile of trajectories

Table I provides the individual characteristics of groups identified by the model. A history of parental wheezing tended to be more common in the persistent wheezing adolescents (45.5%) compared with other trajectories. Adolescents with persistent wheezing had an atopic dermatitis prevalence of 50.9% at age 5 years and 23.6% at age 13 years, which were higher than other trajectories. The distribution of rhinitis was similar in the groups of ages 5, 9, and 13 years, with a lower rate in class 5 (never/infrequent) and class 1 (early-onset transient) and an increased rate in class 2 (late-onset transient), class 3 (low frequent), and class 4 (persistent). At the age of 13 years, the rhinitis rate was as high as 96.4% in the persistent wheezing group, compared with less than 59.3% in the never/infrequent and the early-onset transient wheezing groups.

**Table I:** Individual characteristics of groups identified by the LCGA

Characteristics	Classes∗				
	Early-onset transient	Late-onset transient	Low frequent	Persistent	Never/infrequent
Sex: female	45.7	51.6	42.6	38.2	56.0
Parental history of wheezing	32.6	25.8	22.0	45.5	16.4
Smoking exposure	44.4	36.7	26.2	38.9	26.5
Low income	9.3	7.1	10.7	10.4	4.0
Pet ownership	6.5	6.7	8.2	3.8	10.4
AD at 5 y	19.6	25.8	23.3	50.9	13.7
AD at 9 y	15.2	12.9	17.0	34.6	12.9
AD at 13 y	8.7	13.3	18.0	23.6	9.3
Rhinitis at 5 y	37.0	56.7	51.7	60.0	30.6
Rhinitis at 9 y	47.8	71.0	64.4	85.5	48.6
Rhinitis at 13 y	58.7	74.2	80.3	96.4	59.3

*AD*, Atopic dermatitis.

All data are presented as percentage.

∗ Five trajectory classes were obtained by using LCGA. Missing values were handled by the full information maximum likelihood using all available information in all observations. The start value was 500.

### Predictor of trajectories

The results of linking group membership to covariates are provided in Table II. Parental history of wheezing was significantly associated with the early-onset transient wheezing class (odds ratio [OR], 3.19; 95% CI, 1.20-8.46) or the persistent wheezing class (OR, 4.66; 95% CI, 2.35-9.26) relative to the reference class, that is, the never/infrequent wheezing class. Smoking exposure was also associated with a higher probability belonging to the early-onset transient wheezing class (OR, 3.03; 95% CI, 1.16-7.89) and the persistent wheezing class (OR, 2.06; 95% CI, 1.01-4.19) compared with the reference class. Female participants had a lower risk of being in the persistent wheezing class compared with male participants, relative to the reference class (OR, 0.45; 95% CI, 0.23-0.88).

**Table II:** ORs for tests of categorical latent variable multinomial logistic regression using the 3-step procedure in Mplus

Classes∗	Complete data set				Multiple imputation†			
	OR∗	95% CI		*P* value	OR∗	95% CI		*P* value
		Lower	Upper			Lower	Upper	
Early-onset transient								
Sex (female vs male)	0.52	0.21	1.33	.172	0.57	0.23	1.42	.229
Parental history of wheeze (yes vs no)	**4.07**	**1.45**	**11.45**	**.008**	**3.19**	**1.20**	**8.46**	**.020**
Smoking exposure (yes vs no)	**2.82**	**1.07**	**7.45**	**.036**	**3.03**	**1.16**	**7.89**	**.023**
Low income (yes vs no)	4.30	0.92	19.97	.063	3.14	0.66	14.97	.151
Pet ownership (yes vs no)	0.22	0.01	9.67	.430	0.38	0.02	6.88	.508
Late-onset transient								
Sex (female vs male)	0.94	0.31	2.81	.911	0.83	0.30	2.31	.724
Parental history of wheeze (yes vs no)	1.77	0.45	6.99	.414	1.75	0.54	5.69	.356
Smoking exposure (yes vs no)	1.04	0.28	3.91	.950	1.58	0.52	4.75	.418
Low income (yes vs no)‡	—	—	—	—	1.58	0.15	17.31	.706
Pet ownership (yes vs no)	0.86	0.12	6.02	.882	0.70	0.10	4.92	.718
Low frequent								
Sex (female vs male)	0.47	0.21	1.02	.054	0.56	0.27	1.17	.120
Parental history of wheeze (yes vs no)	1.25	0.41	3.79	.690	1.33	0.49	3.62	.573
Smoking exposure (yes vs no)	0.98	0.39	2.44	.958	0.95	0.38	2.37	.912
Low income (yes vs no)	**4.45**	**1.08**	**18.28**	**.039**	3.29	0.79	13.82	.103
Pet ownership (yes vs no)	1.05	0.26	4.26	.947	0.84	0.21	3.36	.799
Persistent								
Sex (female vs male)	0.48	0.23	1.00	.051	**0.45**	**0.23**	**0.88**	**.020**
Parental history of wheeze (yes vs no)	**6.10**	**2.83**	**13.15**	**<.001**	**4.66**	**2.35**	**9.26**	**<.001**
Smoking exposure (yes vs no)	**2.42**	**1.10**	**5.30**	**.028**	**2.06**	**1.01**	**4.19**	**.047**
Low income (yes vs no)	**4.06**	**1.05**	**15.69**	**.042**	2.92	0.81	10.53	.101
Pet ownership (yes vs no)	0.40	0.07	2.20	.294	0.36	0.07	1.82	.213

Boldface indicates statistical significance.

∗ Model was fitted using the 3-step procedure in Mplus. The reference class was the never/infrequent wheezing class.

† Multiple imputation was used to deal with missing data on covariates. Variables used for multiple imputation processer including sex, parental history of wheeze, smoking exposure, low income, pet ownership, maternal age, and maternal education level. Fifty data sets were generated to obtain pooled parameters.

‡ The number of low-income individuals assigned to this group was very small because of the deletion of the missing values, resulting in an unusually small covariate coefficient.

### The relationship between wheezing phenotypes and lung function at 13 years

**Supplementary Fig E3:**
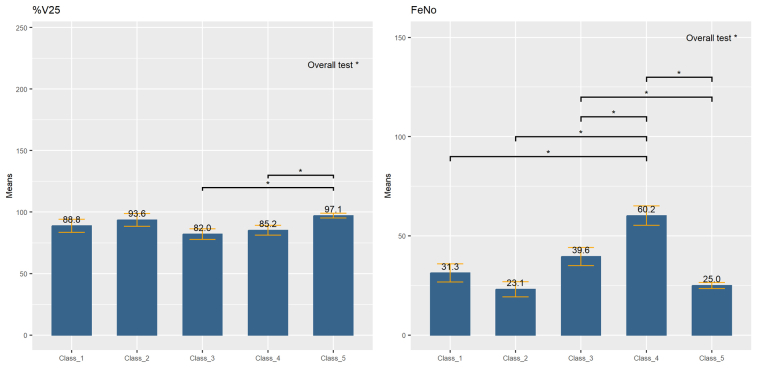


Table III provides the predicted means of lung function at 13 years in each class and the IOS, spirometry, and Feno using the BCH procedure in Mplus. The overall test found significant variation between the groups for %V25 (adjusted *P* = .035) and Feno (adjusted *P* = .01). Further comparisons across groups for %V25 and Feno are plotted in Fig E3 (in the Online Repository available at www.jaci-global.org). The paired-wise test found a significant difference (adjusted *P* < 05). %V25 was significantly lower in class 4 (persistent) than in class 5 (never/infrequent). The Feno level was significantly higher in class 4 (persistent) than in class 5 (never/infrequent).The airway resistance variables evaluated by IOS were similar across the groups (Table III).

**Table III:** Predicted means of lung function at 13 y in each class and overall tests using the BCH procedure in Mplus

Lung test	Parameter	Class 1 (early-onset transient)		Class 2 (late-onset transient)		Class 3 (low frequent)		Class 4 (persistent)		Class 5 (never/infrequent)		Adjusted *P* value†
		Mean∗	SE∗	Mean∗	SE∗	Mean∗	SE∗	Mean∗	SE∗	Mean∗	SE∗	
IOS	TV	0.61	0.04	0.54	0.04	0.65	0.05	0.60	0.03	0.63	0.02	.523
	Z at 5 Hz	0.36	0.02	0.40	0.02	0.37	0.02	0.39	0.02	0.38	0.01	.806
	R at 5 Hz	0.35	0.02	0.38	0.02	0.36	0.01	0.38	0.01	0.36	0.01	.834
	R at 20 Hz	0.30	0.01	0.32	0.02	0.30	0.01	0.32	0.01	0.32	0.01	.906
	R5-R20	0.05	0.01	0.06	0.02	0.05	0.01	0.06	0.01	0.05	0.00	.774
	X at 5 Hz	−0.09	0.01	−0.09	0.01	−0.09	0.01	−0.10	0.01	−0.10	0.00	.982
	Resonant frequency (logarithmic)	2.40	0.07	2.41	0.08	2.38	0.06	2.45	0.05	2.34	0.02	.473
	AX (logarithmic)	−1.49	0.18	−1.51	0.24	−1.63	0.18	−1.35	0.13	−1.58	0.06	.806
	Asthma Intellig‡	4.53	0.25	5.13	0.36	4.79	0.22	5.07	0.23	4.73	0.11	.704
	Rex at 5 Hz	0.35	0.02	0.41	0.03	0.38	0.02	0.39	0.02	0.38	0.01	.726
	Rin at 5 Hz	0.33	0.02	0.33	0.03	0.34	0.02	0.34	0.02	0.33	0.01	.984
	Xex at 5 Hz	−0.09	0.01	−0.10	0.01	−0.09	0.01	−0.10	0.01	−0.10	0.00	.870
	Xin at 5 Hz	−0.10	0.01	−0.09	0.01	−0.09	0.01	−0.10	0.01	−0.09	0.00	.984
Spirometry	FVC	2.56	0.11	2.41	0.12	2.48	0.09	2.54	0.07	2.52	0.03	.906
	%FVC	79.89	1.78	80.22	2.91	78.55	2.07	81.84	1.47	81.69	0.79	.806
	FEV_1_	2.34	0.09	2.22	0.11	2.21	0.08	2.29	0.07	2.32	0.03	.806
	%FEV_1_	83.90	1.98	84.28	2.97	80.74	2.06	84.54	1.69	85.89	0.84	.480
	FEV_1_G§	91.68	1.14	92.00	1.35	90.09	1.11	90.08	1.04	92.06	0.44	.548
	%FEV_1_G‖	104.52	1.29	104.67	1.55	102.73	1.28	102.79	1.18	104.64	0.51	.774
	MMF	2.96	0.15	2.82	0.17	2.71	0.12	2.78	0.12	3.04	0.05	.113
	%MMF	94.69	4.28	93.81	5.02	87.74	3.65	90.30	3.25	99.19	1.60	.064
	PEF	4.54	0.21	3.97	0.29	4.32	0.19	4.44	0.15	4.33	0.07	.810
	%PEF	83.44	3.12	77.37	5.01	80.32	3.24	83.72	2.57	82.05	1.22	.870
	V50	3.25	0.16	3.07	0.19	3.01	0.13	3.04	0.12	3.32	0.06	.167
	%V50	83.40	3.80	82.67	4.70	78.14	3.23	79.49	2.71	87.02	1.41	.108
	V25	1.80	0.11	1.81	0.11	1.63	0.09	1.70	0.09	1.92	0.04	.074
	%V25	**88.82**	**5.28**	**93.62**	**5.23**	**81.99**	**4.26**	**85.19**	**3.99**	**97.11**	**1.97**	**.035**
	ExtrapV	0.11	0.01	0.14	0.01	0.11	0.01	0.12	0.01	0.13	0.00	.421
	ExtrapV%	4.43	0.31	5.99	0.46	4.49	0.30	4.88	0.36	5.04	0.12	.223
Feno		**31.30**	**4.64**	**23.15**	**3.77**	**39.63**	**4.59**	**60.23**	**4.91**	**25.04**	**1.48**	**.010**

*BCH,* Block-Croon-Hagenarrs; *AX*, area of reactance; *ExtrapV*, extrapolated volume; *FVC*, forced vital capacity; *MMF*, maximal midexpiratory flow; *PEF*, peak expiratory flow; *R*, resistance; *R5-R20*, difference between resistance at 5 and 20 Hz; *Rex*, expiratory resistance; *Rin*, inspiratory resistance; *TV*, tidal volume; *V25*, forced expiratory flow at 25% of FVC; *V50*, forced expiratory flow at 50% of FVC; *X*, reactance; *Xex*, expiratory reactance; *Xin*, inspiratory reactance; *Z*, total impedance. Boldface indicates statistical significance.

∗ The mean and SE shown here were obtained from models fitted by the Bolck-Croon-Hagenaars procedure in Mplus.

† The Benjamini-Hochberg method was used to adjust the *P* values.

‡ Asthma Intellig: The area of the yellow section in the UPG graph (it increases in value when there is resistance in the airways or lungs).

§ Growth-corrected.

‖ Growth-corrected (depending on context).

### The relationship between wheezing phenotypes and IgE sensitization

Table IV provides the predicted probabilities of IgE sensitization at age 13 years in each group. The overall test found significant variation (adjusted *P* < .05) by groups in Bet v 1, Can f 1, Cry j 1, Der f 1, Der f 2, Der p 1, Der p 2, and Fel d 1. Paired-wise test (see Fig E4 in this article’s Online Repository at www.jaci-global.org) found a significant difference (adjusted *P* < .05). Bet v 1, Can f 1, Cri j 1, Der f 1, Der p 1, and Fel d 1 were significantly higher in class 4 (persistent) than in class 5 (never/infrequent).

**Table IV:** Predicted probability of serum allergen-specific IgE at 13 y in each class and overall tests using the DCAT process in Mplus

IgE	Class 1 (early-onset transient)	Class 2 (late-onset transient)	Class 3 (low frequent)	Class 4 (persistent)	Class 5 (never/infrequent)	Adjusted *P* value∗
Aln g 1	18.6	21.3	23.4	24.4	13.6	.439
Amb a 1	12.2	18.4	22.9	33.1	13.9	.117
Ara h 8	13	13.1	17.5	23	11.2	.515
**Bet v 1**	**31.7**	**34.2**	**26.6**	**39.4**	**15.7**	**.009**
Blo t 5	9.7	19.7	9	27.6	8.1	.070
**Can f 1**	**15.6**	**2.7**	**10.5**	**27.4**	**7.8**	**.023**
Cor a 1.0101	20.8	28.4	23.6	22.7	12.2	.188
Cor a 1.0401	23.8	22.8	26.2	30.1	13.9	.144
**Cry j 1**	**62.7**	**75**	**83.6**	**84.9**	**62.1**	**.005**
Cup a 1	50.7	67.8	67.5	65.6	53.3	.238
Cyn d 1	8.7	21.5	19.8	18	14	.656
**Der f 1**	**58.6**	**85.1**	**66.7**	**88.4**	**43.1**	**.005**
**Der f 2**	**55.8**	**77.5**	**59.1**	**89.2**	**34.6**	**.005**
**Der p 1**	**57.2**	**82**	**61.2**	**86.6**	**40.3**	**.005**
**Der p 2**	**55.7**	**77**	**59.3**	**85.8**	**35**	**.005**
**Fel d 1**	**48.7**	**39.5**	**41.2**	**48.8**	**23.2**	**.005**
Gly m 4	14.1	17.4	12.9	25.3	10.1	.264
Hev b 8	13.1	4.2	12.9	16.5	7.1	.421
Jug r 2	9.6	9.5	13.8	11	9.9	.982
Mal d 1	27.8	31.3	24.2	29.9	15.3	.144
Mer a 1	12.7	8.3	10.4	14.8	6.5	.608
Ph1 p 1	15	18.1	20.8	21.9	11.8	.488
Ph1 p 4	15.2	17.9	22.1	18.7	14	.822
Pla a 2	11.6	19.8	10.3	16	14.4	.904
Pru p1	23.7	21.1	25.9	26.4	12.8	.179

Data are presented as probability (%). Boldface indicates statistical significance.

∗ The Benjamini-Hochberg method was used to adjust the *P* values.

## Discussion

This is the first study to describe latent phenotypes of wheezing and their associations with lung function and IgE component sensitization in Japanese adolescents. Of the 5 wheezing trajectories, the persistent wheezing phenotype was associated with higher Feno levels and greater aeroallergen sensitization compared with the never/frequent phenotype. However, no impairment of lung function of IOS parameters, forced vital capacity (FVC), FEV_1_, maximal mid-expiratory flow, and peak expiratory flow was observed across all wheezing phenotypes except %V25 in the persistent wheezing phenotype. The prevalence of wheezing is influenced by environment, ethnicity, and genetic factors.

Early-life wheezing due to viral respiratory infections, particularly respiratory syncytial virus (RSV) and human rhinovirus (HRV), is common and has been linked to childhood asthma. However, whether these infections cause asthma or indicate a predisposition remains controversial.^24^ Respiratory viruses, particularly RSV and HRV, play key roles in the development and exacerbation of obstructive respiratory diseases in children, with RSV serving as an inducer of long-term airway changes and HRV acting as a trigger in predisposed individuals.^25^ Respiratory infections can cause lasting damage to the respiratory system, potentially leading to persistent wheezing. Because this study involves a birth cohort recruited from the general population of children and not from children visiting hospitals, one of the study’s strengths is its applicability to the general population, making the results more generalizable. However, a limitation is that we could not gather detailed data from hospital medical records such as management of respiratory infections, which is a key aspect that could have added more insight.

Our Japanese adolescents in the persistent wheezing phenotype did not show a decline in lung function of IOS parameters, FVC, FEV_1_, maximal midexpiratory flow, and peak expiratory flow except %V25. Our results differed from those of studies^4^, ^5^, ^6^, ^7^, ^8^ in the United States, Europe, and Australia where wheezing phenotypes were associated with a decline in lung function. The ALSPAC birth cohort in the United Kingdom^7^ addressed that persistent wheezing was strongly associated with reduced FEV_1_/FVC ratio and forced expiratory flow (FEF)_25-75_ and elevated Feno levels in adolescents. A Melbourne birth cohort also showed a similar association between persistent wheezing and decreased lung function in adolescents.^6^ The Canadian Asthma Primary Prevention Study likewise found that all wheezing phenotypes were associated with impaired lung function (FEV_1_) in adolescents.^4^ Spirometry in adolescents by a pooled analysis in 5 birth cohorts in the United Kingdom demonstrated that all wheezing phenotypes were associated with reduced lung function.^8^ In Sweden’s BAMSE birth cohort,^5^ all asthma phenotypes were negatively correlated with FEV_1_ in adolescence. Our Japanese adolescents’ wheezing prognosis from birth seems to be unique compared with Western populations. Although throughout history Japan had children with severe asthma and wheezing, the current number of asthma-related hospitalizations has decreased, and there are almost no asthma-related deaths.^3^ Pediatric asthma mortality seems to be nearly eradicated. These findings are consistent with the trends observed in our epidemiologic data. When this cohort study began, inhaled corticosteroids became widely used and long-term management medications for asthma significantly advanced, resulting in better asthma control for many children. Japan’s universal health care system also ensures that all children have access to medical care, including emergency visits and specialists. Because of the widespread availability of medications and the health care system, it is hypothesized that even when wheezing occurs, children could promptly receive appropriate medical treatment, which may have prevented a decline in lung function.

As mentioned earlier, pediatric asthma and wheezing control in daily life has significantly improved in Japan. However, more than half of the adolescents were sensitized to house dust mites or cedar pollen. Similar findings were observed in adolescents in Austria^26^ in whom almost half were allergen-sensitized, but the sensitization patterns differed by geographic location and ethnicity. Therefore, elucidating the relationship between wheezing phenotypes, lung function, and sensitization in an Asian population provides valuable insights highlighting differences from Western populations.

In addition, more than half the adolescents also had concomitant rhinitis. In children, the sharp increase in rhinitis is becoming one of the allergic conditions that need to be addressed in Japan,^27^ replacing asthma as a primary concern. With the increase in rhinitis, there has also been a rise in patients with pollen-food allergy syndrome (PFAS). At age 13 years, 1 in 10 individuals exhibited symptoms of PFAS in our cohort populations.^28^ PFAS is one of the manifestations of the atopic march. Moving forward, measures against PFAS will be necessary alongside addressing allergic rhinitis.^29^

In this study, persistent wheezing was associated with an increase in Feno levels. However, nearly all of these cases also had concomitant rhinitis, and so the rise in Feno levels might also reflect inflammation in the upper airways due to rhinitis rather than airway inflammation.^30^ Further investigation is needed regarding the transition of allergic rhinitis and IgE sensitization into adulthood. In the BAMSE cohort,^31^ remission of rhinitis in adulthood was rarely observed if it was present in childhood. The T-Child cohort will continue to be followed up long-term to evaluate the natural history of childhood wheezing and rhinitis into adulthood.

IgE antibody responses may be a marker of distinct respiratory phenotypes, distinguishing allergic asthma from transient nonatopic viral-induced wheezing. Previous studies^32^ have shown that IgE antibody responses represent multiple distinct atopic susceptibilities rather than a single atopic phenotype, each varying in its association with asthma presence and severity. The persistent nature of asthma, marked by airway hyperresponsiveness and lung function impairment in school-age children, is driven by ongoing allergic airway inflammation that starts within the first 3 years of life. In contrast, children with a nonatopic wheezing phenotype tend to outgrow their symptoms by school age and maintain normal lung function into puberty.^33^ Future research should focus on identifying the distinct mechanisms underlying different atopic susceptibilities and their specific contributions to asthma development and severity. Longitudinal studies are needed to clarify how early-life allergic airway inflammation influences long-term lung function and airway hyperresponsiveness. In addition, investigating the factors that contribute to the resolution of nonatopic wheezing could provide insights into potential intervention strategies. Understanding the interplay between genetic predisposition, environmental exposures, and immune system development will be essential for advancing personalized approaches to asthma prevention and management.

The strength of this study lies in conducting high-quality prospective longitudinal birth cohort study incorporating objective biomarkers such as serum-specific IgE, spirometry, and IOS as variables. There were, however, some limitations in this study. First, loss to follow-up resulted in missing data for several individuals, which is a common limitation in birth cohort studies. The loss of follow-up resulted in only 475 cases left for final analysis, which prevented further paired-wise tests for parts of IgE because of low positive rates. The second limitation was the inability to examine asthma phenotypes on the basis of asthma diagnosis. In Japan, even when episodes of frequent wheezing meet asthma diagnostic criteria, there is a tendency for physicians not to formally diagnose asthma although appropriate asthma treatment is prescribed. Therefore, because physician-diagnosed asthma would likely be an inaccurate outcome measure, evaluating outcomes on the basis of wheezing symptoms was considered the most suitable approach.

This study identified 5 wheezing phenotypes and their associations with adolescents’ lung function and IgE sensitization. Unlike findings from Western cohorts, Japanese adolescents with persistent wheezing throughout childhood did not exhibit significant lung function decline. However, persistent wheezing was associated with elevated Feno levels, which may be attributed to concurrent allergic rhinitis rather than lower airway inflammation. In addition, IgE sensitization to house dust mites and cedar pollen was prevalent, highlighting the need for continued monitoring for development of PFAS in the long-term. Future follow-up into adulthood will provide valuable insights into the trajectory of wheezing phenotypes and allergic conditions.

**Clinical implications**

Regardless of the various wheezing trajectories from birth, adolescents in Tokyo did not experience significant lung function decline. Wheezing phenotypes in adolescents in Tokyo had unique characteristics compared with Western populations.

## Disclosure statement

This study was funded by the 10.13039/100007786National Center for Child Health and Development, Tokyo, Japan.

Disclosure of potential conflict of interest: The authors declare that they have no relevant conflicts of interest.

## Figures and Tables

**Supplementary Fig E4:**