# Supplementary material for: Lung functions and IgE component sensitizations: Five wheezing phenotypes in adolescents from the T-Child study in Tokyo
Source: J Allergy Clin Immunol Glob. 2025 Apr 19;4(3):100480. doi: 10.1016/j.jacig.2025.100480 (PMC12127645; doi:10.1016/j.jacig.2025.100480)
Supplement: Supplementary Data [file mmc1.docx]

**Supplemental Material**

**Title of tables**

**Table E1** Baseline table

**Table** E**2** Comparison between used and unused data

**Table E3** Median and interquartile range for variables of spirometry and impulse oscillometry at 13 years old

**Table E4** Model Fit Indicators

**Table E5** Average posterior probabilities by assigned groups based on maximum posterior probability rule

**Figure legends**

**Figure E1** Flow chart of the study

**Figure E2** Serum allergen-specific IgEs at 13 years (%)

**Figure E3 T**he latent class growth analysis model from one to six groups with quadratic trajectories.

**Figure E4** The paired-wise test for predicted lung function means at 13 years across groups by BCH procedure in Mplus. Class1: Early onset transient; Class2: Late-onset transient; Class 3: Low frequent; Class4: Persistent; Class5: Never/infrequent. %V25: Percentage of Predicted Forced Expiratory Flow at 25% of Forced Vital Capacity; FeNO: Fractional exhaled nitric oxide. * p<0.05. We use the BH (Benjamini & Hochberg) method to adjust the p-values.

**Figure E5** The paired-wise test for predicted the probability of IgE at 13 years across groups by DCAT process in Mplus. Class 1: Early onset transient; Class 2: Late-onset transient; Class 3: Low frequent; Class 4: Persistent; Class 5: Never/infrequent. * p<0.05. We use the BH (Benjamini & Hochberg) method to adjust the p-values.

**Table E1** Baseline table

|  | n | Total | % |
| --- | --- | --- | --- |
| Female | 242 | 475 | 51.0 |
| Parental history of wheeze | 107 | 471 | 22.7 |
| Smoking exposure | 142 | 469 | 30.3 |
| Low income | 27 | 423 | 6.4 |
| Pet ownership | 41 | 469 | 8.7 |
| Wheezing at 1y | 123 | 472 | 26.1 |
| Wheezing at 2y | 128 | 468 | 27.4 |
| Wheezing at 3y | 80 | 454 | 17.6 |
| Wheezing at 4y | 79 | 456 | 17.3 |
| Wheezing at 5y | 92 | 469 | 19.6 |
| Wheezing at 6y | 83 | 458 | 18.1 |
| Wheezing at 7y | 64 | 460 | 13.9 |
| Wheezing at 8y | 51 | 461 | 11.1 |
| Wheezing at 9y | 50 | 471 | 10.6 |
| Wheezing at 11y | 43 | 443 | 9.7 |
| Wheezing at 12y | 29 | 446 | 6.5 |
| Wheezing at 13y | 28 | 473 | 5.9 |
| AD at 5 years | 97 | 470 | 20.6 |
| AD at 9 years | 76 | 471 | 16.1 |
| AD at 13 years | 58 | 472 | 12.3 |
| Rhinitis at 5 years | 183 | 469 | 39.0 |
| Rhinitis at 9 years | 265 | 471 | 56.3 |
| Rhinitis at 13 years | 318 | 473 | 67.2 |

AD: atopic dermatitis.

**Table E2** Comparison between used and unused data

|  |  | Data not used for analysis |  |  | Data used for analysis |  |  |
| --- | --- | --- | --- | --- | --- | --- | --- |
| Variables |  | N | % |  | N | % | p value |
| Sex | Male | 577 | 53.7 |  | 233 | 49.1 | 0.09 |
|  | Female | 498 | 46.3 |  | 242 | 50.9 |  |
| Parental history of wheeze | No | 828 | 78.9 |  | 364 | 77.3 | 0.49 |
|  | Yes | 222 | 21.1 |  | 107 | 22.7 |  |
| Smoking exposure | No | 582 | 62.7 |  | 327 | 69.7 | 0.01 |
|  | Yes | 346 | 37.3 |  | 142 | 30.3 |  |
| Low income | No | 919 | 92.6 |  | 396 | 93.6 | 0.51 |
|  | Yes | 73 | 7.4 |  | 27 | 6.4 |  |
| Pet ownership | No | 812 | 87.7 |  | 428 | 91.3 | 0.05 |
|  | Yes | 114 | 12.3 |  | 41 | 8.7 |  |
| Maternal ages | >=35 years | 661 | 61.5 |  | 263 | 55.5 | 0.03 |
|  | <35 years | 414 | 38.5 |  | 211 | 44.5 |  |
| Low education level of mother | No | 923 | 90 |  | 412 | 90.9 | 0.55 |
|  | Yes | 103 | 10 |  | 41 | 9.1 |  |

**Table E3** Median and interquartile range for variables of spirometry and impulse oscillometry at 13 years old

|  | Median | IQR |
| --- | --- | --- |
| TV | 0.57 | 0.26 |
|  |  |  |
| R at 5 Hz | 0.35 | 0.11 |
| R at 20 Hz | 0.31 | 0.1 |
| R5-R20 | 0.04 | 0.09 |
| X at 5 Hz | -0.1 | 0.05 |
| Resonant frequency | 9.84 | 5.135 |
| AX | 0.22 | 0.255 |
| FVC | 2.49 | 0.605 |
| %FVC | 81.1 | 14.4 |
| FEV1 | 2.31 | 0.555 |
| %FEV | 85.3 | 15.65 |
| MMF | 2.89 | 1.055 |
| %MMF | 93.3 | 30.15 |
| PEF | 4.37 | 1.425 |
| %PEF | 81.3 | 22.9 |
| V50 | 3.19 | 1.15 |
| %V50 | 82.6 | 27 |
| V25 | 1.78 | 0.8 |
| %V25 | 90.6 | 36.75 |
| ExtrapV | 0.11 | 0.05 |
| ExtrapV% | 4.49 | 2.365 |
| FeNo | 22 | 27 |

IQR: interquartile range; TV: Tidal Volume; R at 5 Hz: Resistance at 5 Hz; R at 20 Hz: Resistance at 20 Hz; R5-R20: Difference between Resistance at 5 Hz and 20 Hz; X at 5 Hz: Reactance at 5 Hz; Resonant frequency (Log): Resonant Frequency (Logarithmic); AX (Log): Area of Reactance (Logarithmic); ; FVC: Forced Vital Capacity; %FVC: Percentage of Predicted Forced Vital Capacity; FEV1: Forced Expiratory Volume in 1 second; %FEV1: Percentage of Predicted Forced Expiratory Volume in 1 second;FEV1G: Growth-corrected Forced Expiratory Volume in 1 second; MMF: Maximal Mid-Expiratory Flow; %MMF: Percentage of Predicted Maximal Mid-Expiratory Flow; PEF: Peak Expiratory Flow; %PEF: Percentage of Predicted Peak Expiratory Flow; V50: Forced Expiratory Flow at 50% of Forced Vital Capacity; %V50: Percentage of Predicted Forced Expiratory Flow at 50% of Forced Vital Capacity; V25: Forced Expiratory Flow at 25% of Forced Vital Capacity; %V25: Percentage of Predicted Forced Expiratory Flow at 25% of Forced Vital Capacity; ExtrapV: Extrapolated Volume; ExtrapV%: Percentage of Predicted Extrapolated Volume; FeNO:Fractional exhaled nitric oxide.

**Table E4** Model fit indicators for latent class growth analysis model with 1-6 groups

|  | Number of classes (order of polynomial for each class) | | | | | | |
| --- | --- | --- | --- | --- | --- | --- | --- |
|  | 1 (2) | 2 (2,2) | 3 (2,2,2) | 4 (2,2,2,2) | **5 (2,2,2,2,2)^＃^** | 5 (1,2,2,2,2) | 6(2,2,2,2,2,2) |
| Log likelihood | -2283.21 | -1791.29 | -1740.62 | -1710.91 | **-1693.11** | -1696.75 | -1678.79 |
| Akaike's Information Criterion (AIC) | 4572.42 | 3596.58 | 3503.23 | 3451.82 | **3424.22** | 3429.50 | 3403.59 |
| Bayesian Information Criterion (BIC) | 4584.91 | 3625.73 | 3549.03 | 3514.27 | **3503.32** | 3504.44 | 3499.34 |
| Sample-Size Adjusted BIC (SSABIC) | 4575.39 | 3603.51 | 3514.11 | 3466.66 | **3443.02** | 3447.31 | 3426.35 |
| Entropy | - | 0.931 | 0.82 | 0.82 | **0.79** | 0.81 | 0.82 |
| Vuong-Lo-Mendell-Rubin (VLMR) likelihood ratio test | - | <0.0001 | 0.0005 | 0.0064 | **0.5634** | 0.2162 | 0.06 |
| Lo-Mendell Rubin adjested LRT test (LMR-LRT) | - | <0.0001 | 0.0007 | 0.0075 | **0.5715** | 0.2278 | 0.064 |
| Bootstrapped likelihood ratio test (BLRT) | - | <0.0001 | <0.0001 | <0.0001 | **<0.0001** | <0.0001 | <0.0001 |

^＃^Best fitted model

**Table E5** Average posterior probabilities from latent class growth analysis by assigned groups based on maximum posterior probability rule

|  |  |  | Latent class |  |  |
| --- | --- | --- | --- | --- | --- |
| Most likely latent class membership | 1 | 2 | 3 | 4 | 5 |
| 1 | 0.77 | 0.05 | 0.02 | 0.00 | 0.16 |
| 2 | 0.07 | 0.80 | 0.09 | 0.04 | 0.00 |
| 3 | 0.02 | 0.10 | 0.84 | 0.04 | 0.00 |
| 4 | 0.00 | 0.02 | 0.01 | 0.97 | 0.00 |
| 5 | 0.03 | 0.00 | 0.06 | 0.00 | 0.91 |
